# Bias‐Controlled Quantitative Analysis of Golgi‐Stained Neurons via High‐Fidelity Denoising

**DOI:** 10.1111/cpr.70291

**Published:** 2026-11-14

**Authors:** Shuangshuang Jia, Qiaowei Tang, Binfu Fan, Jichao Zhang, Yanling Xue, Ke Li, Jun Hu, Ying Zhu

**Affiliations:** ^1^ Institute of Materiobiology, College of Sciences, Shanghai University Shanghai China; ^2^ National Center for Translational Medicine (Shanghai) Hefei Branch, Anhui University of Chinese Medicine Hefei China; ^3^ Shanghai Synchrotron Radiation Facility, Shanghai Advanced Research Institute, Chinese Academy of Sciences Shanghai China

**Keywords:** analysis, bias control, Golgi staining, high‐fidelity denoising, neuron

## Abstract

Quantitative analysis of Golgi‐stained neurons is fundamentally limited by non‐specific precipitates that introduce substantial bias into morphological measurements. Conventional denoising approaches, designed mainly for low‐resolution medical imaging or reconstruction artefacts, are insufficient for suppressing this chemically induced noise at sub‐micrometre resolution. Here, we present a high‐fidelity denoising strategy for Golgi‐stained neurons (HiFi Golgi), a bias‐controlled analytical strategy for three‐dimensional (3D) Golgi‐stained neuronal imaging. We established a multidimensional evaluation framework to systematically quantify the trade‐off between noise suppression and structural fidelity, enabling rigorous comparison of candidate filtering strategies. Using this framework, an optimised low‐parameter bilateral filter was identified that minimises noise‐induced bias while preserving neuronal morphology. The HiFi Golgi strategy consistently reduces bias across diverse brain regions, imaging resolutions, and model species without parameter re‐optimization, improving the reliability and reproducibility of single‐neuron reconstruction. This work provides a generalizable and high‐fidelity analytical approach for bias‐controlled volumetric imaging, offering a reproducible framework for quantitative morphological analysis in complex neuronal systems.

## Graphical abstract

A bias‐controlled analytical strategy minimises quantitative bias while preserving neuronal structural fidelity in Golgi‐stained volumetric imaging.

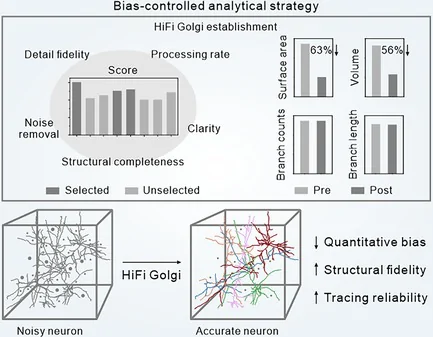

## 1 Introduction

Quantitative analysis of 3D biological imaging data is fundamentally a problem of measurement accuracy and bias control [1, 2, 3, 4]. In high‐resolution volumetric imaging, background signals can introduce substantial systematic errors, leading to biased estimation of structural parameters and compromised reproducibility [5, 6, 7]. These challenges become particularly pronounced when the noise originates from chemical processes rather than imaging or reconstruction artefacts, as such signals often exhibit intensity and spatial characteristics similar to true structures [8].

Golgi staining combined with synchrotron‐based X‐ray imaging enables visualisation of sparse neuronal architectures at sub‐micrometre resolution within large tissue volumes [9, 10, 11, 12, 13, 14]. However, the staining process inevitably generates non‐specific precipitates that are difficult to distinguish from neuronal structures using conventional intensity‐ or threshold‐based criteria [8]. These chemically induced signals are widely distributed and directly compromise quantitative measurements, resulting in bias in key morphological parameters such as branch number and length.

Existing denoising approaches, including spatial‐domain filter, transform‐domain filter, non‐local self similarity prior based methods, and deep based methods [15, 16, 17, 18], have demonstrated effectiveness in medical imaging and reconstruction artefact suppression [19, 20, 21]. Nevertheless, these methods are primarily optimised for acquisition‐related noise [22] and lack explicit consideration of bias introduced by chemically generated signals. More importantly, current strategies typically evaluate denoising performance using visual quality or metrics such as peak signal‐to‐noise ratio [23, 24], without establishing a quantitative framework to assess the trade‐off between noise suppression and structural fidelity. As a result, the impact of denoising on quantitative accuracy remains poorly defined.

Here, we present HiFi Golgi, a bias‐controlled analytical strategy for Golgi‐stained 3D imaging data. By establishing a multidimensional quantitative evaluation framework, we systematically characterise the trade‐off between noise suppression and structural preservation and identify an optimised filtering strategy that minimises systematic bias while maintaining neuronal fidelity. This approach provides a generalizable analytical framework for bias‐controlled quantitative imaging of complex biological systems.

## 2 Results and Discussion

### 2.1 Assessment of Noise‐Induced Bias and Evaluation Framework for Golgi‐Stained Volumetric Imaging

In high‐resolution synchrotron‐based X‐ray imaging, non‐specific precipitates generated during Golgi staining not only degrade image readability but also introduce bias into the quantitative analysis of neuronal morphological parameters. To explicit the impact of noise on quantitative outcomes, we first analysed unprocessed datasets. Mouse brain samples were stained using the high‐pressure Golgi‐Cox (HP Golgi‐Cox) method and imaged at an isotropic voxel resolution of 650 nm, from which a volumetric region of 130 μm × 130 μm × 130 μm was randomly selected from the reconstructed data for subsequent analysis. In the unprocessed data, noise exhibited substantial overlap with true neuronal structures in both grayscale intensity and spatial distribution, thereby significantly compromising the measurement of key parameters such as neuronal branch number and length (Figure S1).

To establish a criteria for balancing noise suppression and neuronal structural fidelity, we constructed a systematic evaluation framework designed to characterise the trade‐off between bias reduction and structural preservation across different bias‐controlled strategies (Figure 1A). This framework evaluates the performance of each strategy using five complementary metrics: clarity and noise removal, which primarily assess background suppression and bias reduction; detail fidelity and structural completeness, which assess the preservation of neuronal morphology; and processing time, which evaluates practical applicability for large‐volume data analysis. The framework therefore provides an initial screening of candidate strategies by jointly considering noise suppression, structural preservation, and analytical efficiency, rather than relying on a single visual criterion. Based on this framework, we selected eight commonly employed spatial‐domain filters as candidate methods, including Bilateral, Box, Despeckle, Gaussian, Majority, Median, symmetric nearest neighbour (SNN), and Recursive filters, and systematically evaluated their suitability for synchrotron‐based X‐ray imaging data of Golgi‐stained brain tissue. For each bias‐controlled strategy, low, median, and high neighbourhood size parameters were applied. 3D visualisation revealed that while most bias‐controlled strategies suppressed background noise to some extent, their performance varied markedly with parameter selection (Figure S2). For instance, Gaussian and SNN filters exhibited only marginal improvements in noise suppression with increasing neighbourhood parameters, whereas Majority and Median filters, despite reducing noise under high parameter settings, concomitantly inflicted pronounced distortions on neuronal morphology (Figure S2). In contrast, the remaining bias‐controlled strategies achieved enhanced noise suppression without discernible structural damage.

**FIGURE 1:**
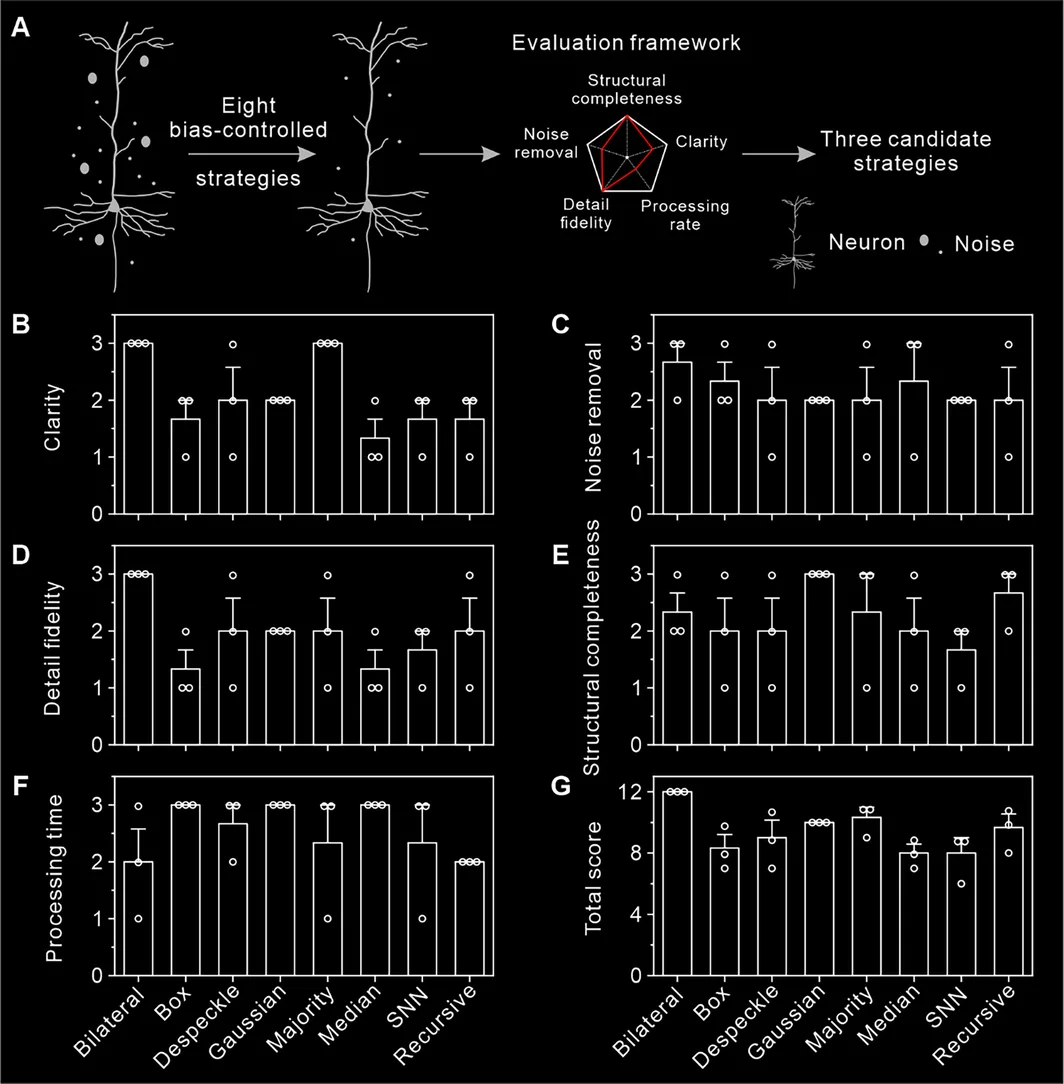
Evaluation framework for Golgi‐stained volumetric imaging. (A) Flowchart of the evaluation framework. The noisy neuronal dataset was processed via eight different bias‐controlled strategies, yielding denoised datasets. The results were then evaluated with five metrics (clarity, noise removal, detail fidelity, structural completeness, and processing time). Based on the scoring results, three candidate bias‐controlled strategies were selected. (B–G) Scores (B–F) and total scores (G) of different bias‐controlled strategies on various metrics (clarity, B; noise removal, C; detail fidelity, D; structural completeness, E; processing time, F). For each strategy, three different parameters (low, median, and high for neighbourhood size) were set. Data are presented as mean + SEM.

Based on the evaluation framework described above, we further employed a standardised scoring approach to comprehensively compare the performance of different bias‐controlled strategies across multidimensional metrics. The evaluation criteria comprised four visual assessment metrics, including clarity, noise removal, detail fidelity, and structural completeness, and one quantitative metric, processing time. Visual metrics were utilised to assess the trade‐off between background suppression and structural fidelity, whereas processing time served to assess practical utility for large‐volume data analysis. For each metric, a score ranging from 1 (weak effect) to 3 (excellent effect) was given based on processing outcomes, and the mean score for each strategy across individual metrics was calculated. Statistical analysis revealed that Bilateral and Majority filters got the highest mean scores for clarity metric (Figure 1B). Bilateral filter demonstrated superior performance in both noise removal and detail fidelity metrics (Figure 1C,D), Gaussian filter received the highest score for structural completeness metric (Figure 1E), and Box, Gaussian, and Median filters exhibited advantages in processing time metric (Figure 1F). These results indicate that different denoising strategies lead to substantially different levels of bias propagation in downstream quantitative analysis. Total mean scores across the five metrics identified Bilateral, Gaussian, and Majority filters as the top three candidates, with composite scores of 12.0, 10.0, and 10.3, respectively, which were subsequently selected for further bias analysis and optimization (Figure 1G). Collectively, these results underscore pronounced differences among bias‐controlled strategies in balancing noise suppression and structural preservation. This systematic screening lays the groundwork for subsequent bias–fidelity trade‐off analysis.

### 2.2 Optimization of Bias‐Controlled Strategy via Bias–Fidelity Tradeoff Analysis

To systematically evaluate the capacity of different bias‐controlled strategies in reducing quantitative bias, the three candidate strategies selected from the preceding screening were subjected to systematic qualitative and quantitative assessment from two dimensions: noise suppression efficacy and neuronal structural fidelity (Figure 2A,B). With respect to noise suppression, 3D visualisations and magnified views of noise regions revealed that in datasets processed with the Majority filter, noise was markedly suppressed as the neighbourhood size increased. Noise levels in the Majority filter with low neighbourhood size (ML) group remained indistinguishable from those in the unprocessed data, whereas in magnified views of the Majority filter with high neighbourhood size (MH) group, discernible noise signals were virtually absent (Figure 2C). The Gaussian filter exhibited minimal parameter dependence in noise suppression (Figure 2D), with overall noise suppression efficacy exceeding that of the Majority filter with median neighbourhood size (MM) group but inferior to that of the MH group. In contrast, the Bilateral filter displayed pronounced parameter dependence in noise suppression. The Bilateral filter with low neighbourhood size (BL) group performed comparably to the MM group and was inferior to the Gaussian filter groups, whereas the Bilateral filter with median neighbourhood size (BM) and Bilateral filter with high neighbourhood size (BH) groups demonstrated substantially enhanced noise suppression capacity, albeit still weaker than that achieved by the MH group (Figure 2E).

**FIGURE 2:**
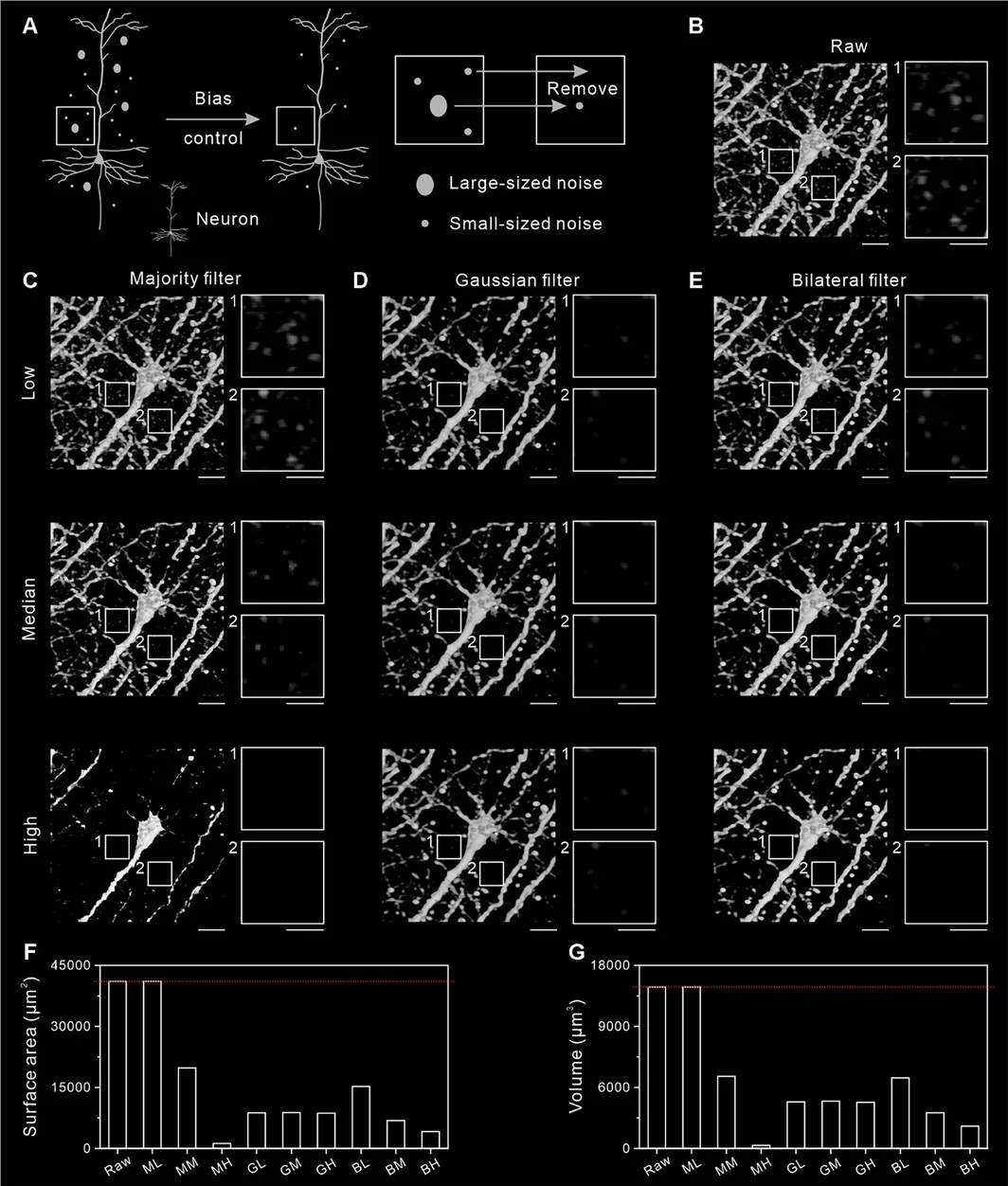
Quantitative analysis of noise suppression by different bias‐controlled strategies. (A) Schematic diagram illustrating noise suppression by the bias‐controlled strategy. After bias control, large‐sized noise was suppressed to smaller ones, and small‐sized noise was removed. (B) The 3D visualisations of a region without bias control and high‐magnification views of the noise. Scale bar: 20 μm (3D visualisations) and 8 μm (high‐magnification views). (C–E) The 3D visualisations of a region processing with different bias‐controlled strategies (Majority filter, C; Gaussian filter, D; Bilateral filter, E) and high‐magnification views of the noise. For each filter, three different parameters (low, median, and high for neighbourhood size) were set. Scale bar: 20 μm (3D visualisations) and 8 μm (high‐magnification views). (F–G) Quantitative analysis of noise surface area (F) and volume (G) before and after bias control.

Subsequently, quantitative analysis of noise surface area and volume was performed. Noise surface area and volume were derived by subtracting the surface area and volume of neuronal structures from the total surface area and volume, respectively. The quantitative results were in close consistent trends with direct visual observations (Figure 2F,G). The Majority filter and Bilateral filter groups exhibited a consistent trend in noise suppression, wherein larger neighbourhood sizes corresponded to more pronounced noise suppression. Notably, noise surface area and volume levels in the ML group remained comparable to those prior to processing, indicating that noise was not effectively removed under this condition. Across the Gaussian filter groups, no obvious differences in noise surface area or volume were observed among different parameter settings, consistent with the qualitative visual assessment. Among these, the MH group exhibited the most effective noise suppression, with noise surface area and volume declining to 3.0% and 1.9% of pre‐processing levels, respectively. Collectively, these results demonstrate that an appropriate bias‐controlled strategy, when combined with suitable neighbourhood size parameters, can effectively suppress non‐specific noise in Golgi‐stained specimens. However, noise suppression alone does not directly translate to improved quantitative accuracy, highlighting the necessity of explicitly evaluating structural fidelity in subsequent analysis.

To address this, the same neuron was extracted for structural fidelity assessment (Figure 3A). We selected the most structurally intact neuron in that region. 3D visualisations and magnified views of fine neuronal structures revealed that in the ML and MM groups, neuronal morphology retained a high degree of similarity to that prior to processing, with neuronal branches and dendritic spines remaining discernible in the magnified views. In contrast, the MH group exhibited severe morphological damage, with the disappearance of neuronal branches and dendritic spine features (Figure 3B,C). Across the Gaussian filter groups, no pronounced differences were observed among different parameter settings, and overall neuronal morphology was largely preserved. However, magnified views revealed fractures in neuronal branches (Figure 3D). Results from the Bilateral filter groups demonstrated that structural fidelity declined as neighbourhood size increased. In the BL group, neuronal branches and dendritic spines were clearly visible, whereas in the BM and BH groups, neuronal branches exhibited fracturing (Figure 3E).

**FIGURE 3:**
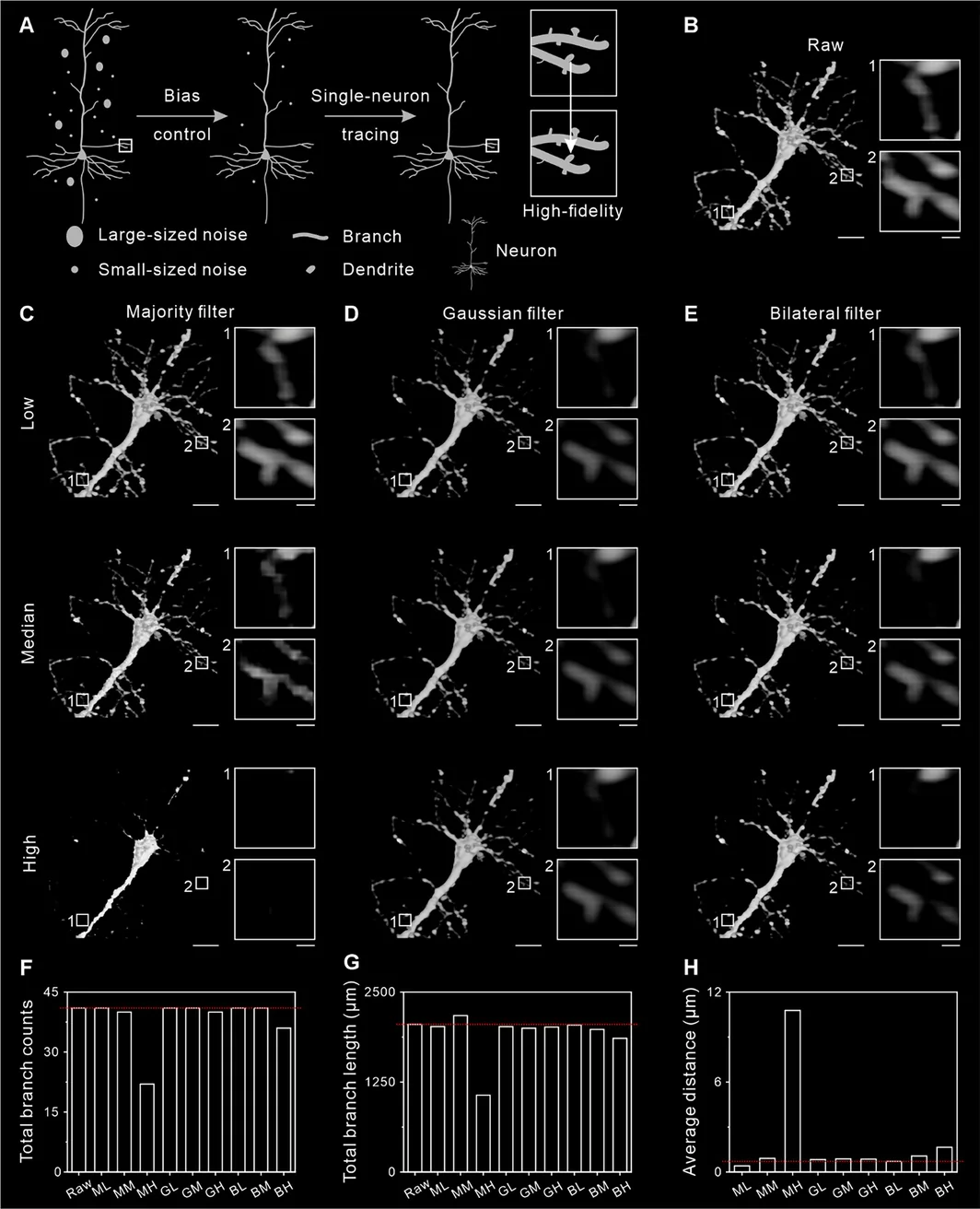
Quantitative analysis of neuronal fidelity by different bias‐controlled strategies. (A) Schematic diagram illustrating neuronal fidelity by bias‐controlled strategies. After bias control, the fine structure of neurons is affected to varying degrees. (B) The 3D visualisation of a region without bias control and high‐magnification views of the fine structure of the neuron. Scale bar: 20 μm (3D visualisation) and 2 μm (high‐magnification views). (C–E) The 3D visualisations of a region processing with different bias‐controlled strategies (Majority filter, C; Gaussian filter, D; Bilateral filter, E) and high‐magnification views of the neuronal fine structure. For each filter, three different parameters (low, median, and high for neighbourhood size) were set. Scale bar: 20 μm (3D visualisations) and 2 μm (high‐magnification views). (F–H) Quantitative analysis of neuronal total branch counts (F), total branch length (G), and average distance to the neuron without processing (H) before and after bias control.

Quantitative analysis of neuronal branch number and branch length was subsequently performed. Total branch counts revealed that the ML, Gaussian filter with low neighbourhood size (GL), Gaussian filter with median neighbourhood size (GM), BL, and BM groups maintained branch numbers consistent with the original neuron, whereas the MM and Gaussian filter with high neighbourhood size (GH) groups exhibited a reduction of one branch. The MH and BH groups showed substantial declines in branch number (Figure 3F). Branch length analysis indicated that the ML, GL, GM, GH, BL, and BM groups preserved branch lengths comparable to the original neuron, with variations not exceeding 5%. In contrast, the MH and BH groups exhibited pronounced reductions in branch length (Figure 3G). Branch length distribution profiles further confirmed that the ML, GL, GM, BL, and BM groups retained high similarity to the original neuron (Figure S3). Notably, the MM group displayed an anomalous increase in branch length, which may be attributable to erroneous branch identification arising from grayscale value alterations. These results demonstrate that excessive noise suppression can introduce additional bias by distorting neuronal structures. Taken together, the combined analysis of noise suppression efficiency and fidelity of neuronal morphological parameters indicated that the Bilateral filter under the low neighbourhood size parameter condition achieved the optimal bias–fidelity trade‐off. Compared with the ML group, the BL group reduced bias introduced by non‐specific precipitates, while relative to high‐parameter conditions, it maintained superior quantitative fidelity of neuronal morphological features. We further performed quantitative analysis of the mean spatial displacement between processed and original neurons to evaluate structural similarity (Figure 3H). For each node on the processed neuron, the nearest distance to any node on the original neuron was computed, and the average of these distances was calculated. Smaller values indicate higher spatial similarity. Results showed that, except for the ML group, which exhibited negligible noise suppression, the BL group yielded the smallest mean spatial displacement, further corroborating the high‐fidelity preservation of neuronal structure achieved by the BL condition. Furthermore, we used a simulated Golgi‐stained dataset to validate the applicability of the Bilateral filter with low neighbourhood size. In this dataset, the neuron was generated from the SWC file of a real Golgi‐stained neuron, enabling a more accurate assessment of quantitative bias. Morphological analysis showed that the branch counts remained unchanged (48 in both cases), the branch lengths changed by less than 1% after processing, and the average spatial displacement of neuronal nodes was 1.00 μm, supporting the quantitative fidelity of this bias‐control strategy (Figure S4). Based on the foregoing bias–fidelity trade‐off analysis, a high‐fidelity bias‐controlled strategy centred on the Bilateral filter under low neighbourhood size parameter was ultimately established and designated the HiFi Golgi method.

### 2.3 Reduction of Quantitative Bias Across Brain Regions and Imaging Resolutions

To validate the bias‐control consistency and reproducibility of the HiFi Golgi method across distinct anatomical regions and imaging resolution conditions, the identical analytical strategy without any parameter re‐optimization was directly applied to datasets acquired from different brain regions of Golgi‐stained mouse brain samples at varying voxel resolutions, to assess its capacity for consistent reduction of quantitative bias.

Two brain regions with markedly distinct structural characteristics, the entorhinal cortex (EcT) and hippocampus (HIP), were selected for analysis. 3D datasets were acquired via synchrotron‐based X‐ray imaging at isotropic voxel resolutions of 650 nm and 325 nm, respectively (Figure 4A). 3D visualisations revealed the pervasive presence of non‐specific precipitates‐induced noise in both datasets, which substantially interfered with the precise analysis of neuronal structures (Figure 4B,C). The HiFi Golgi method was subsequently applied to both datasets to evaluate noise suppression efficacy and neuronal structural fidelity. Magnified views of noise regions demonstrated that noise in both the EcT and HIP datasets was effectively suppressed following processing, with marked reductions in noise volume (Figure 4D,F). We randomly selected and extracted the same neuron from the dataset (indicated by the red arrow) for further evaluation. 3D visualisations confirmed that the processed neurons retained high spatial similarity with pre‐processing neurons (Figure 4E,G). To quantitatively assess the extent of bias reduction, three cubic regions of 100 × 100 × 100 voxels (indicated by red and blue bounding boxes) were selected from each dataset for statistical analysis of noise surface area and volume. Results showed that in the EcT region, noise surface area and volume decreased to 40.9% and 43.7% of pre‐processing levels, respectively (Figure 4H,I), whereas in the HIP region, noise surface area and volume declined to 47.0% and 68.1% of pre‐processing levels, respectively (Figure 4J,K). These findings demonstrate that the HiFi Golgi method achieves consistent noise suppression and quantitative bias reduction across different brain regions. With respect to neuronal structural fidelity, the branch numbers of neurons extracted from both the EcT and HIP regions remained unchanged relative to pre‐processing counts, and variations in branch length were less than 1% (Figure 4L–O). Branch length distribution analysis further revealed high similarity in overall distribution profiles between pre‐ and post‐processing neurons (Figure S5), confirming that the HiFi Golgi method enables high‐fidelity, reproducible quantitative analysis of neuronal morphology across distinct brain regions and resolution conditions.

**FIGURE 4:**
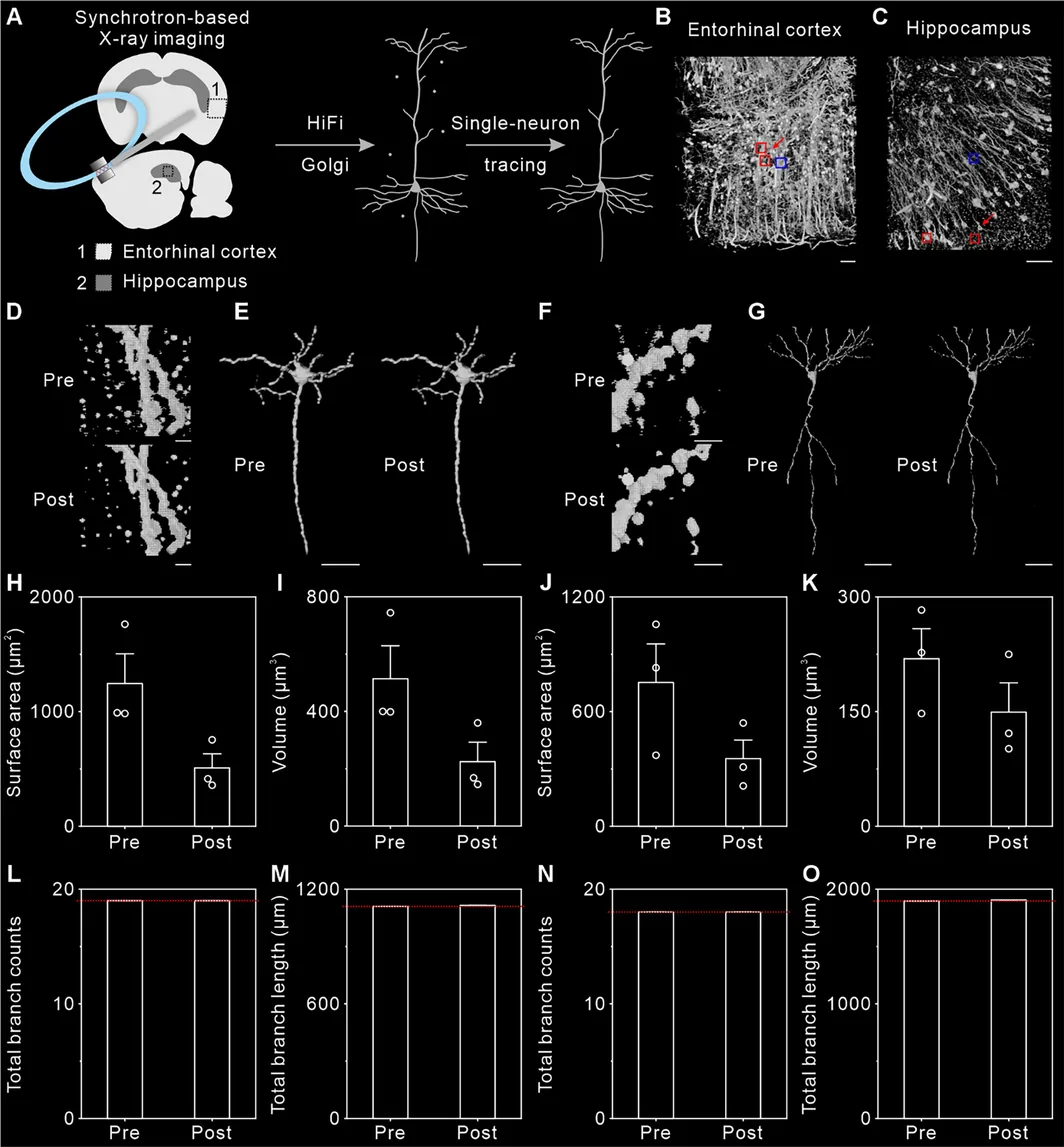
The bias‐control consistency of HiFi Golgi for different brain regions and imaging resolutions. (A) Schematic diagram illustrating the workflow. Mouse brain slices were imaged using synchrotron‐based X‐ray for the EcT and HIP brain regions, followed by HiFi Golgi processing and subsequent single‐neuron tracing and analysis. (B, C) The 3D visualisations of the EcT (B) and HIP (C) brain regions without HiFi Golgi processing. The red and blue boxes indicate the regions for noise analysis, the blue boxes indicate the regions for high‐magnification views, and the red arrows point to the single neurons traced and analysed. Scale bar: 100 μm. (D, F) Images of neuronal structure and noise pre‐ and post‐processing. (D) EcT; (F) HIP. Top: Pre‐; Bottom: Post‐. Scale bar: 10 μm. (E, G) Images of extracted single neuron pre‐ and post‐processing. (E) EcT; (G) HIP. Left: Pre‐; Right: Post‐. Scale bar: 50 μm. (H–K) Quantitative statistics of noise surface area (H, J) and volume (I, K) pre‐ and post‐processing. (H, I) EcT; (J, K) HIP. Data are presented as mean + SEM from *n* = 3 regions. (L–O) Quantitative statistics of single‐neuronal total branch counts (L, N) and length (M, O) pre‐ and post‐processing. (L, M) EcT; (N, O) HIP.

Notably, the HIP dataset was acquired at a higher spatial resolution (325 nm), yet the HiFi Golgi method operated stably without any parameter adjustment under this condition. Moreover, quantitative noise analysis indicated that the method exhibited even more pronounced noise suppression efficacy in the higher‐resolution dataset. This observation further underscores the bias‐control consistency of the analytical strategy to variations in imaging resolution and its ability to achieve consistent reduction of quantitative bias across different imaging scales.

### 2.4 | Bias‐Control Consistency of the Analytical Strategy Across Model Species

To further validate the bias‐control consistency and reproducibility of the HiFi Golgi method under conditions of varying tissue scale and structural complexity, the identical strategy without any modification of analytical parameters was applied to Golgi‐stained brain imaging datasets derived from two distinct model species, SD rat and Bama pig.

Cortical tissue samples from both model species were stained using the high pressure‐assisted Golgi‐Cox (HP Golgi‐Cox) method and subsequently imaged via synchrotron‐based X‐ray tomography at an isotropic voxel resolution of 650 nm to obtain 3D datasets. Original 3D visualisations revealed the abundant presence of non‐specific precipitates‐induced noise signals in both rat and pig brain samples (Figure 5A,B), which posed substantial interference for neuronal morphological analysis. Following processing with the identical HiFi Golgi strategy, magnified views demonstrated that noise was effectively suppressed in brain samples from both model species, with marked reductions in noise volume. Notably, in the pig brain dataset, small‐sized noise was virtually removed (Figure 5C,E). We randomly selected and extracted the same neuron from the dataset (indicated by the red arrow). 3D visualisations confirmed that the processed neurons exhibited high spatial similarity with pre‐processing neurons (Figure 5D,F). Quantitative noise analysis further corroborated this trend. In the cortical region of rat, noise surface area and volume decreased to 41.2% and 49.2% of pre‐processing levels, respectively (Figure 5G,H), whereas in the cortical region of pig, noise surface area and volume declined to 45.4% and 57.8% of pre‐processing levels, respectively (Figure 5I,J). With respect to neuronal structural parameters, branch numbers remained unchanged in both model species, and variations in branch length were less than 2% (Figure 5K–N). Branch length distribution analysis further revealed high similarity in overall distribution profiles between pre‐ and post‐processing neurons (Figures S6 and S7). These results demonstrated that the HiFi Golgi enables high‐fidelity and reproducible quantitative outcomes across species.

**FIGURE 5:**
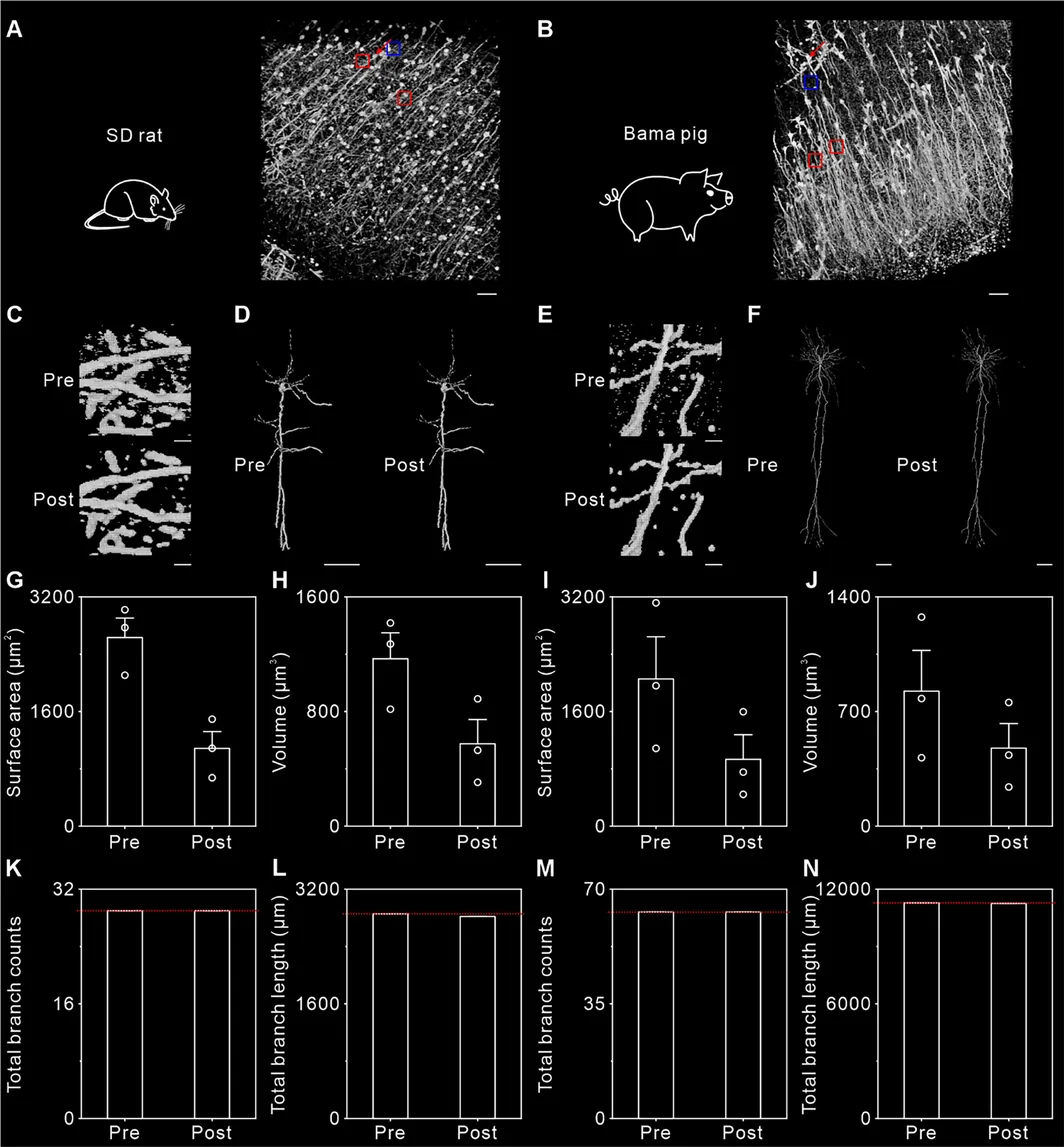
The bias‐control consistency of HiFi Golgi for different model species. (A, B) Schematic diagram and of rat (A) and pig (B) and 3D visualisations of rat (A) and pig (B) brains. The red and blue boxes indicate the regions for noise analysis, the blue boxes indicate the regions for high‐magnification views, and the red arrows point to the single neurons traced and analysed. Scale bar: 100 μm. (C, E) Images of neuronal structure and noise pre‐ and post‐processing. (C) EcT; (E) HIP. Top: Pre‐; Bottom: Post‐. Scale bar: 10 μm. (D, F) Images of extracted single neuron pre‐ and post‐processing. (D) EcT; (F) HIP. Left: Pre‐; Right: Post‐. Scale bar: 50 μm. (G–J) Quantitative statistics of noise surface area (G, I) and volume (H, J) pre‐ and post‐processing. (G, H) EcT; (I, J) HIP. Data are presented as mean + SEM from *n* = 3 regions. (K–N) Quantitative statistics of single‐neuronal total branch counts (K, M) and length (L, N) pre‐ and post‐processing. (K, L) EcT; (M, N) HIP.

Collectively, these results demonstrate that the HiFi Golgi method operates stably across brain samples from distinct model species without parameter re‐optimization, thereby achieving consistent reduction of bias introduced by non‐specific precipitates in Golgi‐stained 3D imaging data. The consistent performance across regions, resolutions, and species suggests that the bias‐control capability of HiFi Golgi is transferable across imaging conditions. Given that Golgi staining is also applicable for postmortem labelling of human brain neurons, the HiFi Golgi method holds considerable promise as a broadly applicable analytical strategy for high‐precision, reproducible quantitative analysis of human neuronal morphology.

### 2.5 | Improved Reliability of Large‐Scale Quantitative Neuronal Reconstruction

We next evaluated the reliability of the established analytical strategy for large‐scale quantitative reconstruction of neuronal morphology. A mouse cortical brain sample stained via the HP Golgi‐Cox method was imaged in the sagittal plane using synchrotron‐based X‐ray tomography at an isotropic voxel resolution of 650 nm. Following 3D reconstruction, the HiFi Golgi method was directly applied to the entire dataset without any parameter modification for bias control, and single‐neuron tracing analysis was subsequently performed (Figure 6A).

**FIGURE 6:**
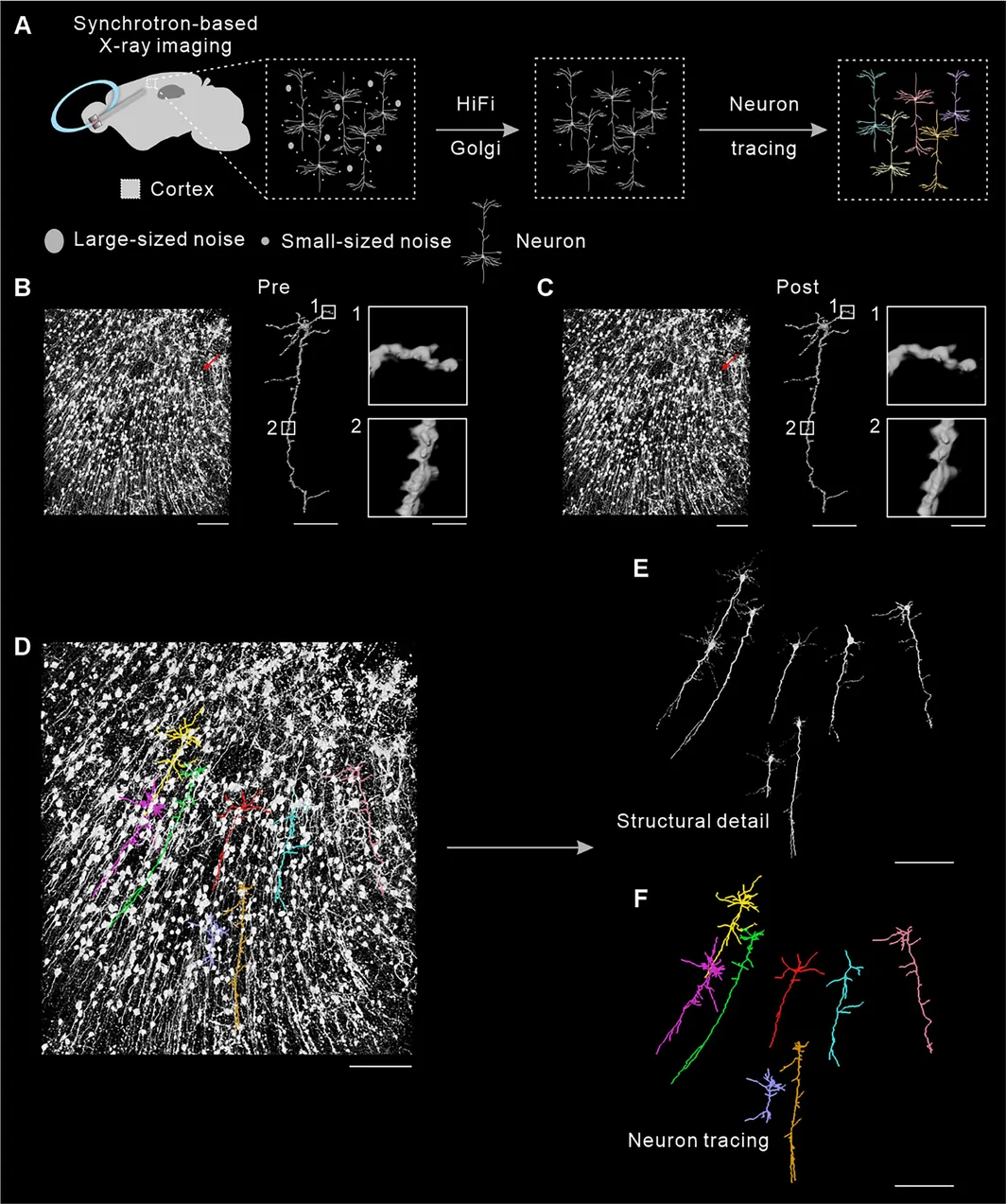
Application of HiFi Golgi for large‐scale neuronal reconstruction. (A) Schematic diagram illustrating the workflow of large‐scale neuronal reconstruction. (B, C) The comparison of the dataset before (B) and after (C) HiFi Golgi processing. The red arrows point to the single neurons traced. Left: The 3D visualisations. Middle: The single neurons. Right: The high‐magnification views. Scale bar: 200 μm (3D visualisations), 100 μm (single neurons) and 10 μm (high‐magnification views). (D) The co‐location of the traced single neurons and the 3D visualisation. Scale bar: 200 μm. (E) The 3D visualisation of the extracted neurons. Scale bar: 200 μm. (F) Large‐scale single neuron tracing. Scale bar: 200 μm.

Comparison of the datasets before and after processing revealed that the HiFi Golgi method markedly reduced background noise levels, thereby rendering neuronal structures more readily discernible within the complex volumetric data (Figure 6B,C). Neurons indicated by arrowheads were subsequently extracted. The images confirmed that the overall morphology of the processed‐neuron remained high similarity to the pre‐processing neuron (Figure 6B,C, middle panels). Magnified views further demonstrated that fine structural features, such as dendritic spines, exhibited no discernible morphological alterations, and neuronal branches remained continuous. No fracturing or modification of branching structures attributable to the analytical strategy was observed (Figure 6B,C, right panels). On this basis, systematic single‐neuron extraction and tracing analysis were conducted for multiple neurons within the dataset (Figures 6D and S8). Representative structural details and corresponding tracing results for eight neurons are shown in Figure 6E,F. This improvement reflects enhanced reliability of quantitative neuronal reconstruction enabled by bias reduction. These findings demonstrate that the HiFi Golgi method is applicable to large‐scale neuronal extraction and tracing analysis.

Collectively, the above results indicate that, owing to the effective balance achieved by the HiFi Golgi method between noise suppression and neuronal morphological fidelity, single‐neuron extraction and subsequent analytical procedures exhibit enhanced consistency and stability in representative samples. This supports the capacity of the analytical strategy to improve the reliability of large‐scale quantitative neuronal morphological reconstruction within complex 3D volumetric imaging data. Given the escalating importance of synchrotron‐based X‐ray tomography for whole‐brain structural imaging, the HiFi Golgi method holds promise for providing a stable and reproducible analytical foundation for the construction of high‐precision brain‐wide neuronal connectivity atlases based on synchrotron X‐ray tomography.

## 3 Conclusions

In summary, this work addresses the critical challenge of bias control in Golgi‐stained synchrotron‐based X‐ray 3D neuronal imaging, where chemically induced non‐specific noise compromises the quantitative characterisation of fine neuronal structures. By establishing a evaluation framework that explicitly captures the trade‐off between noise suppression and structural preservation, we developed HiFi Golgi, a bias‐controlled analytical strategy for volumetric imaging analysis. This approach enables minimization of noise‐induced bias while maintaining neuronal morphological fidelity. Systematic validation demonstrates that HiFi Golgi holds consistency across different brain regions, imaging resolutions, and model species without parameter re‐optimization, achieving consistent bias reduction under diverse conditions. Furthermore, application to large‐scale neuronal reconstruction reveals that bias control directly improves the reliability and consistency of quantitative morphological analysis in complex 3D datasets.

Several limitations should be noted. The current strategy exhibits stable performance at sub‐micrometre resolution. However, its applicability across broader resolutions requires further evaluation. In addition, as a spatial‐domain filtering‐based method, HiFi Golgi primarily reduces noise volume and surface area rather than completely eliminating large non‐specific precipitates, which may still affect structural interpretation under certain conditions. Therefore, although the HiFi Golgi method provides effective noise reduction while preserving neuronal morphology, its denoising capability remains limited compared with advanced deep learning‐based approaches, such as Noise2Void [25, 26]. Future integration of HiFi Golgi with deep learning‐based image restoration strategies may further improve noise suppression, particularly for complex noise patterns, without compromising structural fidelity. Methods such as content‐aware image restoration (CARE) to may also facilitate the recovery of neuronal structures from low‐projection datasets [27]. These approaches could potentially reduce the number of projections required for reliable neuronal reconstruction, enabling shorter acquisition times while maintaining sufficient image quality for synchrotron‐based X‐ray brain imaging.

Given the increasing importance of synchrotron‐based X‐ray imaging for mesoscopic whole‐brain analysis, and the unique suitability of Golgi staining for postmortem human tissue without reliance on viral labelling, HiFi Golgi provides a generalizable, reproducible, and scalable analytical framework for bias‐controlled quantitative imaging. This work offers a practical foundation for high‐precision neuronal morphology analysis and the construction of brain‐wide connectivity maps.

## 4 Materials and Methods

### 4.1 Animals

For mice, C57BL/6 mice (6–8‐week‐old, male) were purchased from Shanghai SLAC Laboratory Animal Co. Ltd., China. For rats, SD rats (6‐week‐old, male) were purchased from Shanghai SLAC Laboratory Animal Co. Ltd., China. For pigs, Bama pigs (2‐month‐old, male) were purchased from Hangzhou tuoshi Biotechnology Co. Ltd., China. The present study was approved by the Ethical Committee of Shanghai Beautiful Life Medica Technology (approval number 009–2014) and the Ethics Committee of Shanghai University (Approval Number: ECSHU 2025–085).

### 4.2 HP Golgi‐Cox Staining

Prior to brain extraction, all model organisms were perfused with 0.9% saline through the heart. Brains were quickly removed from the skull and washed with 0.9% saline to remove blood from the surface. Staining of all brain tissues was performed by the HP Golgi‐Cox method [12].

### 4.3 Synchrotron‐Based X‐Ray Imaging

Data were collected at the BL13HB X‐ray imaging and biomedical application beamline [28] and BL16U2 fast X‐ray imaging beamline [29] of Shanghai synchrotron radiation facility (SSRF) (China). The 900 or 1800 projections were collected over 0°–180° at equal intervals with 325‐ and 650‐nm isotropic voxel resolution and 14‐keV X‐ray energy.

### 4.4 Reconstruction and Visualisation

Projections were reconstructed into tomographic slices using the Gridrec algorithm implemented in phase‐sensitive X‐ray image processing and tomography reconstruction (PITRE) [30] or MOCUPY [31] software. Reconstructed slices were preprocessed in Fiji ImageJ for removal of circular boundaries from micro computed tomography reconstructions and conversion to 8‐bit grayscale format. Volume rendering was performed in Amira using the processed slices.

### 4.5 Bias‐Controlled Strategies

The reconstructed slices were imported into Amira software. The “Image Processing‐Smoothing and Denoising” module was applied to process the images using different filters, with adjustments made to the neighbourhood size. The eight filters applied were: Bilateral filter, Box filter, Despeckle filter, Gaussian filter, Majority filter, Median filter, SNN filter, and Recursive filter. For each bias‐controlled strategy, low, median, and high neighbourhood size parameters were applied. For Bilateral filter, 7, 5, and 3, similarity was set as 20; for Box filter, 5, 4, and 3; for Despeckle filter, 7, 5, and 3; for Gaussian filter, 11, 7, and 3; for Majority filter, 5, 3, and 1; for Median filter, 3, 2, and 1; for SNN filter, 7, 5, and 3; for Recursive filter, 80, 60, and 40.

### 4.6 The Surface Area and Volume of Noise

In Amira software, the “Volume Edit” module was applied to perform volume editing on the dataset, removing noise from the dataset while retaining only the soma and branches of the neurons. Subsequently, “Interactive Thresholding” module was applied to perform threshold segmentation on the dataset before and after noise removal. Then, the “Label Analysis” module was used to statistically analyse the surface area and volume of the dataset before and after noise removal. The surface area and volume of the noise were obtained by subtracting the surface area and volume of the neurons from the total surface area and volume.

### 4.7 Single‐Neuron Extraction, Tracing and Analysis

In Amira software, the “Volume Edit” module was used to perform volume editing on the target neuron, removing noise as well as soma and branches not belonging to the neuron from the dataset, thereby extracting a single neuron. The “Filament Editor” module was then applied to trace the target neuron, and the single‐neuron reconstruction method for Golgi‐stained mouse brain neurons (SNR‐Golgi method) was utilised to repair the branches [14], generating a neuronal pathway file, which was exported in SWC format. Subsequently, Trees toolbox was employed to obtain branch images of the neuron [32], while Python code was used to calculate the total number of branches and total branch length of the neuron. The average distance between nodes of the target neuron and nodes of the original neuron was also computed.

### 4.8 Neuronal Simulation

Neuron simulation was performed using Python code: simulated neurons were generated based on SWC files of real Golgi‐stained neurons, and noise was then added to the simulated neurons to mimic precipitates resulting from non‐specific staining.

### 4.9 Statistical Analysis

#### 4.9.1 Sample Size

For establishment of the HiFi Golgi method, we performed multi‐metric analyses on a single noise region and a single neuron per group. For validation of the HiFi Golgi method, we analysed changes in noise volume and surface area in three independent noise regions per group and performed detailed comparisons of neuronal morphology in one neuron per group. Data were plotted as bar charts using Origin software and were presented as mean + SEM.

## Author Contributions

Y.Z. supervised the research. S.J. and Q.T. performed experiments. B.F. helped in guidance of data analysis. J.Z., Y.X., and K.L. helped in guidance of X‐ray microscopy. J.H. performed critical revisions. S.J., Q.T., and Y.Z. analysed the data and wrote versions of the manuscript. All authors discussed and commented on the manuscript.

## Funding

This work was supported by the National Key Research and Development Program of China (2023YFC3404200, 2023YFC3404203) and National Natural Science Foundation of China (22525605, 22393930, 22393934).

## Conflicts of Interest

The authors declare no conflicts of interest.

## Supporting information


**Figure S1:** 3D visualisation of the dataset and high‐magnification view without bias control. Without bias control, noise exhibited substantial overlap with true neuronal structures in both grayscale intensity and spatial distribution, thereby significantly compromising the measurement of key parameters such as neuronal branch number and length. Scale bar: 200 μm (left) and 20 μm (right).
**Figure S2:** 3D visualisations of datasets processed with different bias‐controlled strategies and neighbourhood sizes. (A–H) 3D visualisation of the dataset (130 μm × 130 μm × 130 μm) processed with different bias‐controlled strategies, including Bilateral filter (A), Box filter (B), Despeckle filter (C), Gaussian filter (D), Majority filter (E), Median filter (F), SNN filter (G) and Recursive filter (H). For each filter, three different parameters (low, medium and high for neighbourhood size) were set.
**Figure S3:** Length distribution of neuronal branches for traced neuron in cortex region. For a 130 μm × 130 μm × 130 μm region, three bias‐controlled strategies (Majority filter, first row; Gaussian filter, second row; and Bilateral filter, third row) were applied, each with high, medium, and low neighbourhood size settings to process the region. Subsequently, neurons were extracted and traced. Neuronal branch length distribution plots were then obtained using Trees toolbox.
**Figure S4:** Simulated neuron processing outcomes using the HiFi Golgi method. (A) 3D visualisation of the neuronal SWC file. (B) 3D visualisation of the simulated neuron. (C) 3D visualisation of the simulated neuron and noise. Left: visualisation of the whole dataset. Right: high‐magnification view of the noise. (D) Quantitative statistics of single‐neuronal total branch counts and length pre‐ and post‐processing. (E, F) Length distribution of neuronal branches pre‐ (E) and post (F) ‐processing.
**Figure S5:** Length distribution of neuronal branches for traced neurons in EcT and HIP region. The HiFi Golgi method was applied to process the datasets from the EcT and HIP brain regions, respectively. Single neurons were then extracted and traced, and their branch length distribution plots were obtained using Trees toolbox.
**Figure S6:** Length distribution of neuronal branches for traced rat neurons. The HiFi Golgi method was applied to process the dataset from the rat brain. Single neurons were then extracted and traced, and their branch length distribution plots were obtained using Trees toolbox.
**Figure S7:** Length distribution of neuronal branches for traced pig neurons. The HiFi Golgi method was applied to process the dataset from the pig brain. Single neurons were then extracted and traced, and their branch length distribution plots were obtained using Trees toolbox.
**Figure S8:** Large‐scale neuronal extraction and tracing. The HiFi Golgi method was directly applied to the entire mouse brain dataset without any parameter modification for bias control. On this basis, systematic single‐neuron extraction and tracing analysis were conducted for multiple neurons within the dataset. Figure S8 presented the structural details and tracing results for these eight neurons. Scale bar: 100 μm.

## Data Availability

The data that support the findings of this study are available from the corresponding author upon reasonable request. The source code for this study is available on GitHub at: https://github.com/QW‐Tang/HiFi‐Golgi.git.
