# Supplementary material for: Bias‐Controlled Quantitative Analysis of Golgi‐Stained Neurons via High‐Fidelity Denoising
Source: Cell Prolif. 2026 Nov 14:e70291. Online ahead of print. doi: 10.1111/cpr.70291 (PMC13768875; doi:10.1111/cpr.70291)
Supplement: Supplementary file 1 [file CPR-9999-e70291-s001.docx]

**Supporting information**

**Bias-controlled quantitative analysis of Golgi-stained neurons via high-fidelity denoising**

Shuangshuang Jia^1,2†^, Qiaowei Tang^1†^, Binfu Fan^3^, Jichao Zhang^3^, Yanling Xue^3^, Ke Li^3^, Jun Hu^1^, Ying Zhu^1,2*^

^1^Institute of Materiobiology, College of Sciences, Shanghai University, Shanghai 200444, China.

^2^National Center for Translational Medicine (Shanghai) Hefei Branch, Anhui University of Chinese Medicine, Hefei, Anhui 230012, China.

^3^Shanghai Synchrotron Radiation Facility, Shanghai Advanced Research Institute, Chinese Academy of Sciences, Shanghai 201204, China.

*E-mail: zhuying331@shu.edu.cn

**Supporting Information:**

**Supporting Figures S1-S8.**

**Supplementary Figures**

**Figure S1.** 3D visualization of the dataset and high-magnification view without bias control.

Without bias control, noise exhibited substantial overlap with true neuronal structures in both grayscale intensity and spatial distribution, thereby significantly compromising the measurement of key parameters such as neuronal branch number and length. Scale bar: 200 μm (left) and 20 μm (right).

**Figure S2.** 3D visualizations of datasets processed with different bias-controlled strategies and neighborhood sizes.

(A–H) 3D visualization of the dataset (130 μm × 130 μm × 130 μm) processed with different bias-controlled strategies, including Bilateral filter (A), Box filter (B), Despeckle filter (C), Gaussian filter (D), Majority filter (E), Median filter (F), SNN filter (G) and Recursive filter (H). For each filter, three different parameters (low, medium and high for neighborhood size) were set.

**Figure S3.** Length distribution of neuronal branches for traced neuron in cortex region.

For a 130 μm × 130 μm × 130 μm region, three bias-controlled strategies (Majority filter, first row; Gaussian filter, second row; and Bilateral filter, third row) were applied, each with high, medium, and low neighborhood size settings to process the region. Subsequently, neurons were extracted and traced. Neuronal branch length distribution plots were then obtained using Trees toolbox.

**Figure S4.** Simulated neuron processing outcomes using the HiFi Golgi method.

(A) 3D visualization of the neuronal SWC file.

(B) 3D visualization of the simulated neuron.

(C) 3D visualization of the simulated neuron and noise. Left: visualization of the whole dataset. Right: high-magnification view of the noise.

(D) Quantitative statistics of single-neuronal total branch counts and length pre- and post-processing.

(E,F) Length distribution of neuronal branches pre- (E) and post (F) -processing.

**Figure S5.** Length distribution of neuronal branches for traced neurons in EcT and HIP region.

The HiFi Golgi method was applied to process the datasets from the EcT and HIP brain regions, respectively. Single neurons were then extracted and traced, and their branch length distribution plots were obtained using Trees toolbox.

**Figure S6.** Length distribution of neuronal branches for traced rat neurons.

The HiFi Golgi method was applied to process the dataset from the rat brain. Single neurons were then extracted and traced, and their branch length distribution plots were obtained using Trees toolbox.

**Figure S7.** Length distribution of neuronal branches for traced pig neurons.

The HiFi Golgi method was applied to process the dataset from the pig brain. Single neurons were then extracted and traced, and their branch length distribution plots were obtained using Trees toolbox.

**Figure S8.** Large-scale neuronal extraction and tracing.

The HiFi Golgi method was directly applied to the entire mouse brain dataset without any parameter modification for bias control. On this basis, systematic single-neuron extraction and tracing analysis were conducted for multiple neurons within the dataset. Figure S8 presented the structural details and tracing results for these eight neurons. Scale bar: 100 μm.
